# A Quantitative Pharmacology Model of Exosome-Mediated Drug Efflux and Perturbation-Induced Synergy

**DOI:** 10.3390/pharmaceutics13070997

**Published:** 2021-06-30

**Authors:** Jin Wang, Bertrand Z. Yeung, M. Guillaume Wientjes, Minjian Cui, Cody J. Peer, Ze Lu, William D. Figg, Sukyung Woo, Jessie L.-S. Au

**Affiliations:** 1 Department of Pharmaceutical Sciences, College of Pharmacy, The University of Oklahoma Health Sciences Center, Oklahoma City, OK 73117, USA; jwang@i-qsp.org (J.W.); byeung@optimumtx.com (B.Z.Y.); minjian-cui@ouhsc.edu (M.C.); 2 Institute of Quantitative Systems Pharmacology, Carlsbad, CA 92008, USA; gwientjes@i-qsp.org; 3 Optimum Therapeutics LLC, Carlsbad, CA 92008, USA; zlu@optimumtx.com; 4 Clinical Pharmacology Program, National Cancer Institute, NIH, Bethesda, MD 20892, USA; cody.peer@nih.gov (C.J.P.); figgw@mail.nih.gov (W.D.F.); 5 College of Pharmacy, Taipei Medical University, Taipei 11031, Taiwan

**Keywords:** quantitative pharmacology model, exosome-mediated drug efflux, chemosensitization mechanism

## Abstract

Exosomes, naturally occurring vesicles secreted by cells, are undergoing development as drug carriers. We used experimental and computational studies to investigate the kinetics of intracellular exosome processing and exosome-mediated drug efflux and the effects of exosome inhibition. The experiments used four human-breast or ovarian cancer cells, a cytotoxic drug paclitaxel (PTX), two exosome inhibitors (omeprazole (OME), which inhibits exosome release, and GW4869 (GW), which inhibits synthesis of sphingolipid ceramide required for exosome formation), LC-MS/MS analysis of PTX levels in exosomes, and confocal microscopic study of endocytic transport (monitored using fluorescent nanoparticles and endocytic organelle markers). In all four cells, exosome production was enhanced by PTX but diminished by OME or GW (*p* < 0.05); the PTX enhancement was completely reversed by OME or GW. Co-treatment with OME or GW simultaneously reduced PTX amount in exosomes and increased PTX amount and cytotoxicity in exosome-donor cells (corresponding to >2-fold synergy as indicated by curve shift and uncertainty envelope analyses). This synergy is consistent with the previous reports that OME co-administration significantly enhances the taxane activity in tumor-bearing mice and in patients with triple negative metastatic breast cancer. The experimental results were used to develop a quantitative pharmacology model; model simulations revealed the different effects of the two exosome inhibitors on intracellular PTX processing and subcellular distribution.

## 1. Introduction

Exosomes are naturally occurring nano-lipid carriers secreted by cells (30–150 nm diameter), have the same membrane as the parent cell, contain endogenous materials (small molecules, proteins, nucleic acids), mediate cell–cell communication and nutrient delivery, circulate in body fluids, and readily enter cells [1,2]. Exosomes can have diverse biological functions [3]. For example, exosomes derived from cancer cells are involved in distal metastatic niche initiation [4,5], intercellular communications (e.g., during drug resistance development [6,7]), and immune system modulation [8,9]. We recently demonstrated that exosome is an intercellular drug transfer mechanism with pharmacological consequences [10]. We and other investigators have shown that exosomes represent an efflux and chemoresistance mechanism for cytotoxic, e.g., paclitaxel, cisplatin, and doxorubicin [10,11,12,13].

Exosomes present unique and favorable properties such as stability, low immunogenicity, and capability of crossing blood-brain-barrier, and there have been steadily increasing interests in engineering and using exosomes as carriers of therapeutics [14,15,16]. For example, exosomes loaded with small molecule drugs such as chemotherapy or kinase inhibitors and with nucleic acids such as siRNA and microRNA have been applied to target multiple tumor types including breast, pancreatic, and glioblastoma cancer [17,18,19].

The formation and intracellular processing of exosomes follows the general scheme of endocytosis [20,21]. Endocytic vesicles are internalized and vesicles fuse with each other to form a larger vesicle or early endosomes (EE). EE is the sorting center where the internalized materials or cellular contents are sorted into fast or slow recycling endosomes, which return the contents, e.g., membrane receptor, to cell membrane. EE can evolve into multivesicular bodies (MVB) that proceed to either the acidified late endosomes (LE, pH 5–6) and then to the enzyme-rich lysosomes (pH 4.5–5) or migrate to a pericellular location where their contents are released as exosomes. A prevailing model is that, during endosomal maturation, parts of EE form tubular structures that become endosome-recycling compartment (ERC), whereas the remaining main body becomes MVB.

The present study used experimental and computational studies to investigate (a) the kinetics of intracellular processing of exosomes and exosome-mediated drug efflux and (b) the effects of perturbing the sorting or release of exosomes on pharmacological outcome. A quantitative pharmacology (QP) model, inclusive of intracellular exosome processing and drug pharmacokinetics and pharmacodynamics, was developed and the experimental results were used to obtain the necessary model parameters. The model was then used to simulate the effects of interfering various processes of the exosome lifecycle on the concentrations in those difficult-to-measure intracellular compartments. Briefly, the experiments were performed using four human cancer cell lines (breast or ovarian) and two exosome inhibitors, *N*,*N*′-Bis[4-(4,5-dihydro-1H-imidazol-2-yl)phenyl]-3,3′-p-phenylene-bis-acrylamide dihydrochloride (GW) and omeprazole (OME). GW inhibits synthesis of sphingolipid ceramide required for the inward budding of endosome lumen to form exosome [22] and causes dose-dependent reduction of exosome production in vitro [23,24]. OME is a proton pump inhibitor that inhibits exosome release [25,26]. The processing and release of exosomes from cells were studied by measuring the concentrations of paclitaxel (PTX) which, after entering a cell, is sorted and released via exosomes [10] and by monitoring the exosomes released into the extracellular fluid using live cell confocal microscopy. Changes of the drug levels in whole cells and exosomes over time were analyzed using a high-performance liquid chromatograph-tandem mass spectrometry and were used together with the drug-induced cytotoxicity data to obtain the model parameter values including the sorting of PTX into exosomes and the release of exosomes. The experimental results show that OME and GW reduced the exosome release along with reduced PTX efflux and produced synergistic cytotoxicity with PTX. Additional QP model simulations indicate that while both inhibition of the formation or the release of exosomes reduced the PTX amount in exosomes, the two inhibitors had different effects on intracellular PTX processing and distribution in subcellular compartments.

## 2. Materials and Methods

Overview. The present investigation used a combination of computational and experimental studies. QP model parameter values were obtained from the experimental results. The established model was then used to simulate the effects of perturbing different steps in exosome-mediated drug efflux on the drug concentrations in different intracellular and extracellular compartments.

In this report, exosome-producing cells are denoted as Donor cells, exosomes collected from drug-treated cells are EXO_drug_, and drug-naïve cells treated with EXO_drug_ are Recipient cells. All concentration terms are bracketed (e.g., [PTX_cell,free_] is concentration of intracellular free PTX). Subscripts are used to denote location (extracellular, intracellular, exosomes) and drug moieties with respect to binding or association (free, bound) with macromolecules or organelles (tubulin, vesicles, exosomes). Subscripts are used to denote the various drug entities and their locations. For example, PTX_medium,free_ and PTX_cell,free_ are free (unbound) drug in medium and cells, respectively; PTX_tubulin_ and PTX_ves_ are intracellular tubulin- and vesicle-bound PTX, and PTX_exo_ is PTX in exosomes, respectively.

QP modeling to quantify difference between inhibiting formation and release of exosomes. We previously developed several computational models to describe the cellular pharmacokinetic (PK) and pharmacodynamic (PD) of PTX [10,27,28,29]. These earlier models accounted for drug transport into and out of cells (including passive diffusion, active efflux via Pgp and exosomes, re-uptake of exosomes via receptor-mediated endocytosis), and the resulting cytotoxicity. The model in the current study is an extension that included the inhibition of exosome formation and release. The model assumptions, equations, and parameter values are shown in Supplementary Materials Table S1. Most model parameters were obtained from our earlier publications, whereas α and β (the extents of inhibition of PTX-sorting into exosomes and release of PTX-loaded exosomes) were obtained as the best-fitting values. For model validation, we used the complete model (with the best-fitting α and β values) to simulate the cytotoxicity in exosome-donor cells (*Cyto_donor_*) of PTX and compared the model-simulated results with additionally obtained PK/PD datasets in Donor cells. The validated model was then used to simulate several intracellular PTX entities that could not be measured experimentally due to technological limitations, as a mean to quantify the effect of inhibiting PTX-sorting into the pre-exosome vesicles vs. inhibiting exosome release. Note the sum of the concentrations of three intracellular entities ([PTX_cell,free_], [PTX_tubulin_], [PTX_ves_]) equaled the total concentration that was experimentally measured as [PTX_donor-lysate_]. PTX in the extracellular fluid comprises three entities, i.e., PTX_medium,free_, macromolecule-bound (PTX_medium,bound_), and PTX_exo_. [PTX_medium,total_] is the PTX concentration added to the culture medium in the beginning of the experiment.

The model-building PK/PD datasets included (a) [PTX_exo_] and [PTX_donor-lysate_] after drug treatments at different [PTX_medium,total_] and different times (300 or 1000 nM [PTX_medium,total_], 8 or 24 h, without or with OME or GW pretreatment); and (b) *Cyto_donor_* at 12 and 48 h after treatment with 0.1 to 1000 nM PTX without or with OME or GW pretreatment. The model-validation datasets included *Cyto_donor_* for 24, 72 or 96 h treatments with single agent PTX and combinations of PTX with OME or GW pretreatment.

Model simulations and data fitting (nonlinear least-squares algorithm) were performed using Matlab Simbiology (Release 2018a, Mathworks, Natick, MA, USA). In order to improve model robustness and predictive reliability, we used only parameter values that could simultaneously fit the datasets in excess of the number of model parameters. The final model was selected by Bayesian information criterion [30], and the best-fitting parameter estimates were obtained using the weighted least-squares non-linear regression.

Reagents and cell culture. PTX, OME, GW, and cell culture grade ethanol were purchased from Sigma-Aldrich (St. Louis, MO, USA). Stock solutions of drugs were prepared in ethanol. PTX concentrations (0.1–1000 nM) were selected to reflect the clinically relevant and achievable range. OME concentrations (3–300 µM) and GW concentrations (1–100 µM) were selected based in part on earlier reports [22,23,24,25]. Four human cancer cell lines were studied in order to reach a broadly applicable conclusion. Human breast adenocarcinoma MCF7 cells were purchased from ATCC (Manassas, VA, USA). LM2 cells, a highly lung-metastatic subline of human breast cancer MDA-MB-231 cells [31], were a gift from Dr. Y. Kang (Princeton University, Princeton, NJ, USA). Human ovarian cancer A2780 and OVCAR4 cells were gifts from Dr. Danny N. Dhanasekaran (University of Oklahoma Health Sciences Center, Oklahoma City, OK, USA). LM2 cells were maintained in DMEM (Mediatech, Manassas, VA, USA), and MCF7, A2780, and OVCAR4 cells in RPMI-1640 (ATCC). The medium for cell growth (Growth Medium) was supplemented with 10% fetal bovine serum (FBS, Atlanta Biologicals, Flowery Branch, GA, USA), whereas the medium for exosome isolation (Conditioned Medium) was supplemented with 10% exosome-depleted FBS (System Biosciences, Palo Alto, CA, USA). All mediums contained 100 IU/mL penicillin and 100 μg/mL streptomycin (Mediatech). Cells were cultured in a humidified incubator at 37 °C in 5% CO_2_.

Exosome isolation, characterization, and quantification. Exosome isolation was performed as previously described [10]. Briefly, cells were cultured with or without drugs, and the resulting Conditioned Medium was collected at preselected time points, centrifuged to remove dead cells and debris. The supernatant was transferred and centrifuged at 100,000× *g* for 18 h at 4 °C. The resulting pellet was washed once with phosphate-buffered saline (PBS), centrifuged at 100,000× *g* for 70 min at 4 °C, and re-suspended in PBS. An aliquot of exosome suspension was used to determine the total protein amount using BCA assay (Pierce kit, ThermoFisher Scientific Inc., Carlsbad, CA, USA). A second aliquot was analyzed for acetylcholinesterase (AChE), an enzyme present in exosomes, using Ellman colorimetric assay. Briefly, exosome suspension was incubated with an aqueous solution containing 1 mM 5,5′-dithio-bis(2-nitrobenzoic acid) and 3 mM acetylthiocholine chloride (substrate for AChE) (Sigma-Aldrich) in a 96-well plate for 40 min at room temperature, and the absorbance at 412 nm was determined using a Synergy HT microplate reader (BioTek Instruments, Winooski, VT, USA). Standard curves of AChE activity were established using MCF7 exosome standards calibrated by NanoSight analysis (System BioSciences), and used to calculate exosome quantity. Exosomes used for biological activity determination were similarly obtained except the post-PBS-washed pellet, which was re-suspended in exosome-free Growth Medium.

Quantification of PTX in exosomes. PTX concentrations in lysates of Donor cells ([PTX_donor-lysate_]) and in EXO_drug_ ([PTX_exo_]) were measured using liquid chromatography–tandem mass spectrometry (LC-MS/MS) [10]. Briefly, PTX was extracted from the sample using methyl t-butyl ether, with docetaxel as the internal standard. The extract was reconstituted with 0.1% aqueous formic acid/methanol (40/60, *v*/*v*), injected onto a Symmetry Shield RP18 column, using a gradient elution scheme of 0.1% formic acid in methanol (increasing methanol from 40% to 100% in 7 min before returning to 40% in another 10 min). The column eluent was directed into an electrospray ionization triple quadrupole mass spectrometer (Micromass Quattro Premier XE; Waters, Milford, MA, USA) for detection based on multiple reaction monitoring of a structural fragment in the positive ion mode, i.e., PTX by the mass transition of m/z 854.0 → 287.0 (collision energy 16 V) and docetaxel by m/z 806.8 → 526.3 (collision energy 9 V). Universal mass spectrometer settings included capillary voltage of 3500 V, cone voltage of 25 V, source temperature 120 °C, desolvation temperature 400 °C, and desolvation gas flow (N_2_) of 600 L/h. A calibration curve covering a dynamic range of 1–1000 ng/mL was constructed, and the best-fitting line was obtained using linear regression with 1/x^2^ weighting. Quality control standards were prepared in quintuplet at each of three levels (low (3 ng/mL), mid (50 ng/mL), high (800 ng/mL)), and back-calculated against the best-fitting line to ensure accuracy and precision of the assay. This assay had sufficient sensitivity to detect PTX_exo_ from cells treated with [PTX_medium,total_] of ≥300 nM.

Drug treatment condition and cytotoxicity measurement. Cytotoxicity in exosome-donor cells (*Cyto_donor_*) of drugs (PTX, OME, and GW) and cytotoxicity in exosome-recipient cells (*Cyto_recip_*) were measured using the sulforhodamine B (SRB) colorimetric assay [10,27]. Briefly, cells were seeded, allowed to attach overnight, treated with drugs, fixed with trichloroacetic acid, washed three times, air-dried overnight, and stained with SRB at room temperature. After washing off the unbound/excess dye, the cell-bound SRB was dissolved in Tris buffer and the absorbance measured at 510 nm using Synergy HT microplate reader. Pilot studies in MCF7 cells indicated 24 h treatment with OME or GW at concentrations up to 868 µM or 300 µM, respectively, and did not cause appreciable cytotoxicity (<5%), whereas prolonged treatment with OME for 96 h produced a minor effect in MCF7 cells (observed maximal drug-induced cytotoxicity (*E_obs,max_*) of 8.2% at between ~9–900 µM drug concentrations). Subsequent studies used OME and GW at fixed concentrations (29 and 10 µM, respectively). For cytotoxicity measurements, Donor cells were pretreated for 24 h with OME or GW followed by PTX for up to 96 h, whereas Recipient cells were treated with EXO_drug_ for 48 h.

Analysis of drug interactivity. Drug interactivity analysis used two methods (curve shift and uncertainty envelope (UE)). Curve shift is a graphical method to enable visualization of the shift in the concentration-response (C-E) curve [32,33,34]. UE is its extension to enable the quantification of the extent of drug interactivity and the analysis of the statistical significance of the interactivity. The envelope in UE is constructed based on the experimental C-E data (mean and variations) of single agents; additivity, synergy, and antagonism are indicated when the observed C-E for drug combinations (*C-E_comb,observed_*) is located within the envelope, outside its left boundary, and outside its right boundary, respectively. The UE method requires definition of *E_obs,max_* and concentration causing 50% *E_obs,max_* (*EC_50_*). Because single agent OME or GW had no cytotoxicity, we arbitrarily set *E_obs,max_* to 1% and *EC_50_* to 100 µM in the analysis. Extents of drug interactivity (EI) were calculated as previously described; EI values of less than 1 indicate synergy [32,33,34]. We did not use the combination index method because it does not provide statistical analysis [35,36].

Time-lapse live cell laser confocal microscopy and quantitative image analysis to study endocytic transport. We used a cationic siRNA-liposome complex (Lipoplex) to study drug effects on exocytosis of exosomes; siRNA was fluorescent-labeled. Lipoplex preparation used the dry film and extrusion method as previously described [37]. Briefly, four lipids (1,2-dioleoyl-3-trimethylammoniumpropane or DOTAP, cholesterol, 1,2-dioleoyl-sn-glycero-3-phosphoethanolamine, and 1,2-distearoyl-sn-glycero-3-phosphoethanolamine-N-[methoxy(polyethylene glycol)-2000, at a molar ratio of 50:30:19:1) were mixed and dissolved in chloroform. Evaporation of chloroform under vacuum yielded a thin lipid film that was subsequently hydrated with RNase-free water (1 mL per 10 mg lipids) at 50 °C for 30 min. The resulting pegylated cationic liposome suspension was passed through an extruder with a 100 nm membrane (Whatman, UK). Lipoplex was formed by gently mixing liposomes with an aqueous solution of siRNA at room temperature, in a 4:1 DOTAP-to-siRNA charge ratio, for at least 20 min before use. The siRNA was directed at metadherin and was conjugated with a fluorescent probe AF647 (pseudo blue fluorescence, Integrated DNA Technologies).

MCF7 cells (5 × 10^5^) were seeded on a µ-Slide I Luer 0.8 slide (Ibidi, WI) and allowed to attach overnight at 37 °C, 5% CO_2_ and 95% humidity in an incubator. Cells were then transduced for 16 h in Growth Medium, with an insect virus (baculovirus) using a human promoter to express red fluorescence protein (RFP) fused with Rab5 (CellLight Reagents, ThermoFisher Scientific) and green fluorescence protein (GFP) fused with Rab7, a marker for late endosomes. After replacing the medium with Lipoplex-containing Growth Medium, the slide was moved to the environmental chamber in SP8 confocal microscope (Leica, Wetzlar, Germany) equipped with 4 solid state lasers. The respective excitation wavelengths for GFP-Rab7, RFP-Rab5, and AF647 were 488, 552, and 638 nm, and the respective emission wavelength ranges were 490–537, 557–632, and 643–765 nm. Time-lapse images with z-stack images covering the bottom-to-top of each cell were taken every 10 min at 1000 Hz (1024 × 1024 resolution) for 6 h or before significant photobleaching occurred. The results were monitored using quantitative image analysis. Briefly, a Macro plug-in script was written to automatically run the multi-step fluorescence signal quantification through ImageJ (National Institutes of Health, Bethesda, MD, USA) in batches (see Supplementary Materials). Because the confocal microscopic images comprised merged RGB signals, it was necessary to first split the images into two channels (red for Rab5, blue for siRNA). Thresholds determined using untreated controls were 30 for Rab5 and 12 for siRNA; images with signals exceeding these threshold values were saved and analyzed using the Image Calculator command that identifies the co-localized red and blue pixels. Areas containing ≥ 4 pixels (to distinguish from random debris) were selected using the Particle Analysis command as regions of interest. The average red and blue fluorescence intensities within individual region were determined and the multiplication product of area and intensity, which provided a measure of the amount, was recorded. To adjust for sample-to-sample variations and potential photobleaching, the results were normalized by the blue fluorescence intensity.

Data and statistical analysis. Experimental results were analyzed for statistical significance using Student’s t-test (paired or unpaired, two-tailed) or Dunnett’s multiple comparisons test with Prism 7 (GraphPad Software, San Diego, CA, USA) or repeated measures ANOVA with SAS 9.0 (SAS Institute, Cary, NC, USA). Model parameter values were obtained by fitting the equations to the experimental results. A *p* value of less than 5% was considered significant. All results are presented as mean ± SEM (standard error of the mean).

## 3. Results

**Figure 1:**
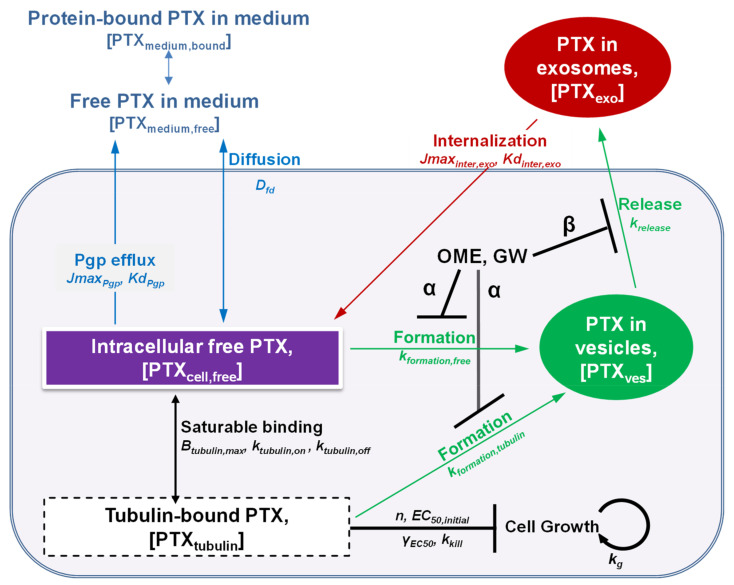
A quantitative model of cellular PTX PK and PD. We extended our previous cellular PK/PD model [10] to capture the inhibition of exosome production/sorting and release by OME and GW. *D_fd_* is rate constant of passive diffusion of free drug; *Jmax_pgp_* is the maximum Pgp-mediated drug efflux rate; *Kd_pgp_* is dissociation constant of drug from Pgp; [PTX_exo_] and [PTX_ves_] are concentration in exosomes and intracellular vesicles, respectively; *Jmax_inter,exo_* is maximum rate of membrane receptor-mediated internalization of exosomes; *Kd_inter,exo_* is dissociation constant from membrane receptor; *k_formation,free_* or *k_formation,tubulin_* and *k_release_* are first-order rate constants for sorting [PTX_cell,free_] or [PTX_tubulin_] into intracellular vesicles and for exosome release, respectively; *B_tubulin,max_* is maximum available drug binding sites in tubulin; *k_tubulin,on_* and *k_tubulin,off_* are rate constants of drug association and disassociation with/from tubulin, respectively. For the PD model, *k_kill_* is the maximal rate constant of cell kill; *EC_50_* is the [PTX_tubulin_] needed to generate 50% of *k_kill_*; *EC_50,initial_* is the *EC_50_* value at time zero, and *γ_EC50_* is the rate of *EC_50_* change per unit time; *n* is Hill exponent; *k_g_* is cell growth rate constant. In view of the negligible intracellular non-saturable PTX binding relative to the tubulin binding, the model excluded non-saturable binding.

Figure 1 shows the QP model structure and the model parameters. The following sections summarize the experimental results.

### 3.1. Effects of PTX, OME, and GW on Exosome Production/Excretion

Table 1 shows the AChE/exosome quantification results. In all four cancer cell lines (two human breast (MCF7, LM2) and two human ovarian (A2780, OVCAR4)), treatment with PTX (100 nM) for 24 h enhanced the extracellular exosome levels (~50%); this is consistent with our earlier finding [10]. On the contrary, pretreatment with OME (29 µM) for 24 h significantly reduced the exosome levels (~30%) in breast cancer cells but had no effects on the ovarian cancer cells, whereas GW (10 µM) pretreatment reduced the exosome levels in all four cells but slightly less in the two ovarian cells. In addition, OME or GW pretreatment completely abolished the PTX-stimulated exocytosis in all four cells; in most cases, the combination groups showed similar exosome levels as for single agent OME or GW.

### 3.2. Effects of PTX and OME on Endocytic Transport

We used a fluorescent (siRNA-labeled) Lipoplex to study drug effects on exocytosis of exosomes. Due to its size (~110 nm in water and 130 nm in culture medium containing 10% fetal bovine serum), Lipoplex are expected to enter cells mainly via receptor-mediated endocytosis and undergo intracellular processing by endocytic organelles [38]. In endocytosis, nano-size particles are internalized and become a part of early endosomes (EE). The contents in EE are sorted into fast or slow recycling endosomes, which return the contents (e.g., membrane receptor) to cell membrane or evolve into multivesicular bodies that migrate to a pericellular location where its contents are released as exosomes [3,39]. The marker for EE is Ras-related protein Rab5, which is also found in exosomes [40]. Occasionally, nanoparticles can enter cells by fluid phase endocytosis, e.g., via macropinosomes, which also express Rab5 [41]. Hence, the appearance of extracellular vesicles with co-localized fluorescent Rab5 and siRNA signals is indicative of exocytosis of the internalized Lipoplex. Note that multivesicular bodies can undergo another pathway to become the acidified late endosomes, which have a different marker (i.e., Rab7).

**Figure 2:**
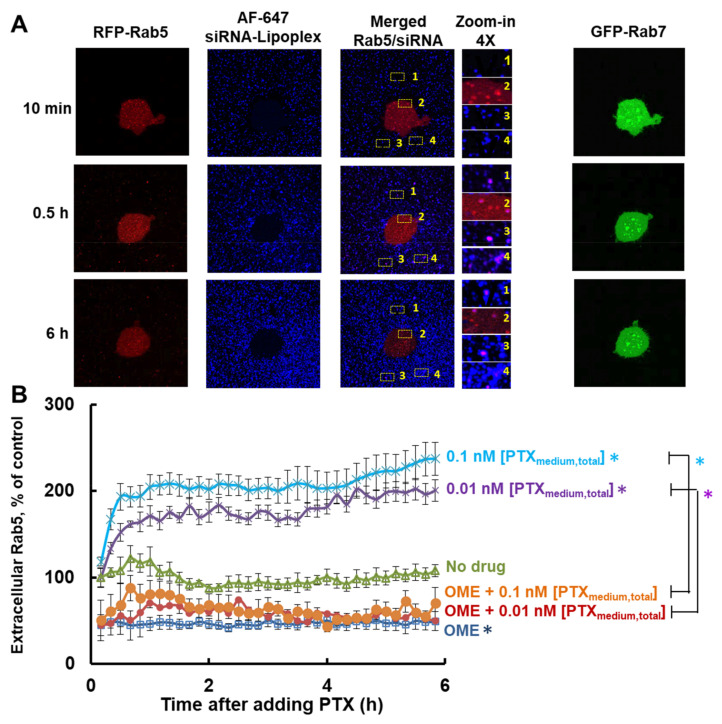
Time-lapse live cell confocal microscopy. RFP-Rab5- and GFP-Rab7-transduced MCF7 cells were treated with OME (29 µM or 10 µg/mL, 24 h), PTX (at [PTX_medium,total_] of 0.01 or 0.1 nM, 6 h), or their combination (OME followed by PTX). Note the RFP-Rab5 or GFP-Rab7 transduction, in addition to highlighting the early or late endosomes, also produced faint diffused red or green fluorescence, respectively, throughout the cell (including the extended pods). Live cell confocal images were taken every 10 min for 6 h after adding PTX (*n* = 6 cells per group). The two control groups were without drug treatment or without OME pretreatment. The vision field of each image is 20 × 20 µm^2^. (**A**) Left 4 panels: representative confocal microscopic images showing vesicles containing both RFP-Rab5 (red) and AF647-siRNA (pseudo blue) in both intracellular and extracellular fluid (images taken at 2 h). Right panel: images of GFP-Rab7 (green) and AF647-siRNA. Note all intracellular or extracellular siRNA signals co-localized with Rab5 and none with Rab7. (**B**) Quantitative image analysis results. * *p* < 0.05 for the difference between three groups (single agent PTX or OME, or no drug control), and between the groups receiving single agent PTX (0.01 and 0.1 nM [PTX_medium,total_]) and their corresponding combination with OME (repeated measures ANOVA). No statistically significant difference between single agent OME and OME + PTX groups (0.01 or 0.1 nM [PTX_medium,total_]) (*p* = 0.56 or *p* = 0.47, respectively; repeated measures ANOVA). Mean ± SEM (6 experiments, duplicate samples per experiment).

Figure 2A shows the live cell confocal microscopy images of fluorescent siRNA/Lipoplex in Rab5/Rab7-transduced MCF7 cells. At early times, i.e., 10 min, the siRNA/Lipoplex signals resided nearly exclusively in extracellular fluid, whereas Rab5 resided only intracellularly. The intracellular siRNA/Lipoplex and the extracellular Rab5 then increased over time. Note all extracellular Rab5 signals co-localized with the siRNA/Lipoplex signals. By contrast, all Rab7 signals remained inside the cell. These results confirmed Lipoplex was internalized into endosomes and then released as exosomes and, hence, could be used to study drug-induced changes in exosome formation and/or release. Figure 2B shows the results obtained in MCF7 cells using PTX (at non-cytotoxic concentrations of 0.01–0.1 nM, which caused <3% *Cyto_donor_*) and OME, alone and in combination. In the absence of drug treatment, the level of extracellular exosomes showed <27% fluctuation over 6 h, suggesting an apparent steady state. Consistent with the AChE/exosome quantification results (Table 1), treatment with non-cytotoxic PTX rapidly increased the exosome levels (within minutes) in a concentration-dependent manner, single agent OME significantly reduced the exosome levels, and OME pretreatment abolished the PTX-stimulated exosomes. These microscopy and imaging results confirm that PTX and OME altered the endocytic transport processes including exosome exocytosis.

### 3.3. OME and GW Pretreatment Altered PTX Efflux

Pretreatment with OME (29 µM) or GW (10 µM) for 24 h significantly elevated [PTX_donor-lysate_] and significantly decreased [PTX_exo_] in cells treated with 1000 nM [PTX_medium,total_] for 24 h (Table 2). While the extents of changes varied among the four cell lines, GW was generally more effective than OME. We next compared the changes in MCF7 cells treated with 300 or 1000 nM PTX for 8 or 24 h, and did not observe apparent concentration- or time-dependent differences.

### 3.4. OME and GW Pretreatment Altered Cyto_donor_ of PTX and Cyto_recip_ of Exosomes Collected from Donor Cells Treated with a Drug (EXO_drug_)

**Figure 3:**
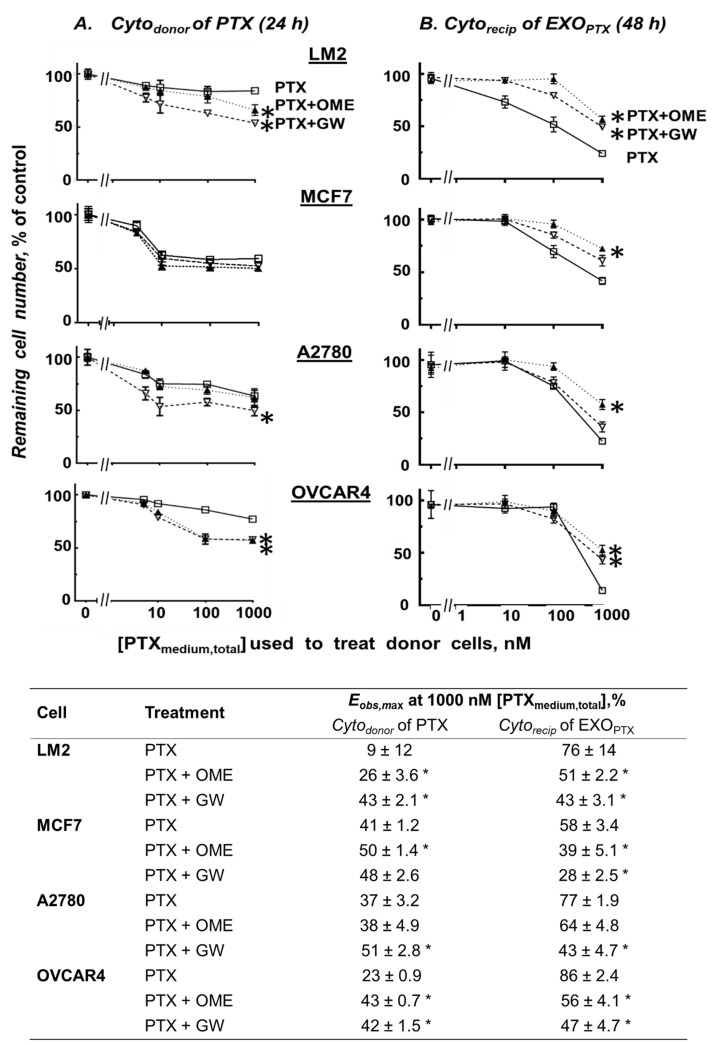
Effects of OME or GW pretreatment on *Cyto_donor_* of PTX and *Cyto_recip_* of EXO_PTX_. Exosome-donor cells were either treated with single agent OME (29 µM), GW (10 µM), or PTX (0.1 to 1000 nM [PTX_medium,total_]) for 24 h, or their combinations. *Cyto_donor_* was measured after pretreating cells with OME or GW for 24 h followed by PTX for additional 24 h (**A**), whereas *Cyto_recip_* was measured after treating cells with EXO_PTX_ collected from Donor cells for 48 h (**B**). Cytotoxicity was measured using SRB assay. *Cyto_donor_* is expressed as % of the no-treatment control group, and *Cyto_recip_* is expressed as % of control group treated with exosomes collected from drug-free Donor cells. Mean ± SEM (*n* = 4 experiments with duplicate samples). Treatments were single agent PTX (squares), PTX + OME (solid triangles), PTX + GW (open triangles). Single agent OME or GW had no appreciable *Cyto_donor_* or *Cyto_recip_* (not shown). *Cyto_donor_* of PTX, without or with OME/GW, reached or approached *E_obs,max_* at or below the highest [PTX_medium,total_] of 1000 nM whereas *Cyto_recip_* of EXO_PTX_ did not reach *E_obs,max_*. The *E_obs,max_* values summarized in the table are the maximal cytotoxicity attained at 1000 nM [PTX_medium,total_]; the higher values for *Cyto_recip_* compared with *Cyto_donor_* was due to the longer treatment duration (48 vs. 24 h). * *p* < 0.05 for the difference between OME + PTX or GW + PTX vs. single agent PTX (1-way ANOVA Dunnett’s multiple comparison).

Figure 3A shows plots of *Cyto_donor_* of PTX (24 h treatment, ±pretreatment with OME or GW for 24 h) vs. [PTX_medium,total_]. *Cyto_donor_* of PTX in all 4 cells depended on [PTX_medium,total_] and were enhanced by addition of OME or GW (e.g., see the tabulated results obtained at 1000 nM [PTX_medium,total_]).

Figure 3B shows plots of *Cyto_recip_* of EXO_drug_ collected from Donor cells treated with PTX at different [PTX_medium,total_] and OME (29 µM) or GW (10 µM), alone or in combination. In all 4 cells, EXO_OME_ or EXO_GW_ had no significant *Cyto_recip_* (<5%, not shown). In contrast, EXO_PTX_ showed appreciable effects (e.g., *E_obs,max_* of 58–86% with 1000 nM [PTX_medium,total_]), which were reduced by OME or GW pretreatment (to 28 or 47%, respectively).

### 3.5. Analysis of Drug Interactivity

**Figure 4:**
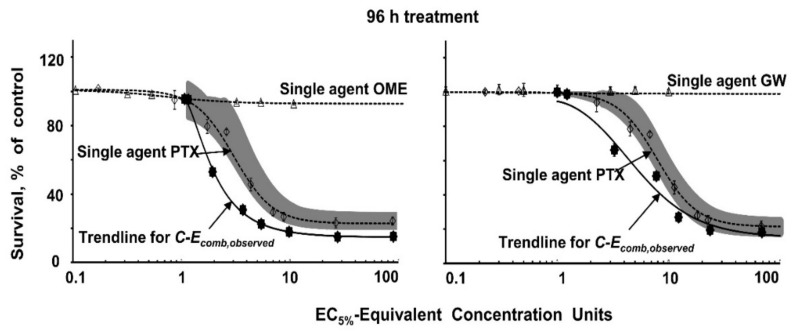
Analysis of drug interactivity. The nature and extent of drug interactivity between OME/GW and PTX was evaluated in MCF7 cells. Cells were pretreated with OME (29 µM) or GW (10 µM) for 24 h, followed by treatment with PTX at various concentrations for 12 to 96 h. The C-E curves for the 96 h drug treatments are shown; single agent OME or GW (open triangles), single agent PTX (open diamonds), and combinations (PTX + OME or PTX + GW, solid dots). Concentration is expressed in EC_5%_ units (1 unit equals 27 µM OME or 1.16 nM [PTX_medium,total_]) to allow plotting of single agents and combinations in a single graph. Lines are best-fitting Hill equation functions for the single agents (dotted lines) or the combinations (solid lines). C-*E_comb,observed_* is C-E curve for the observed results of OME/GW + PTX; C-*E_comb,observed_* located to the left or below the single agent lines indicates a synergistic interaction per Curve Shift analysis. UE (shaded area) indicates the region where synergy or antagonism cannot be concluded at 5% significance, whereas C-*E_comb,observed_* located beyond this region indicates significant synergy (i.e., to the left of or below) or significant antagonism (to the right or above).

*Cyto_donor_* data in MCF7 cells were analyzed to determine if OME or GW synergistically enhanced the PTX activity. Results of curve shift analysis showed a trend of increasing synergistic (leftward) shift of C-E curves of OME + PTX or GW + PTX combinations in Donor cells, compared with single agent PTX; the concentration and time thresholds for the shift were 1–5 nM PTX and ≥12 h. Figure 4 shows the example of 96 h treatment. Results of UE analysis showed the drug interactivity changed from additivity at short treatments (12 and 24 h) to synergy at longer treatments (48–96 h; the 96 h results are shown in Figure 4). EI values were calculated at 50% cytotoxicity and hence could not be attained for the 12 and 24 h treatments that yielded <50% *E_obs,max_*. After treatment for 48, 72, and 96 h, EI values were 0.26, 0.47, and 0.31 for the OME + PTX combinations, and 0.42, 0.76, and 0.54 for the GW + PTX combinations, respectively. The EI values for 96 h treatments corresponded to a ~3-fold synergy for the OME + PTX combination (equals 1 divided by EI of 0.31) and a ~2-fold synergy for the PTX + GW combination (1 divided by EI of 0.52).

### 3.6. Quantitative Pharmacology Model and Evaluation of Model Performance

**Figure 5:**
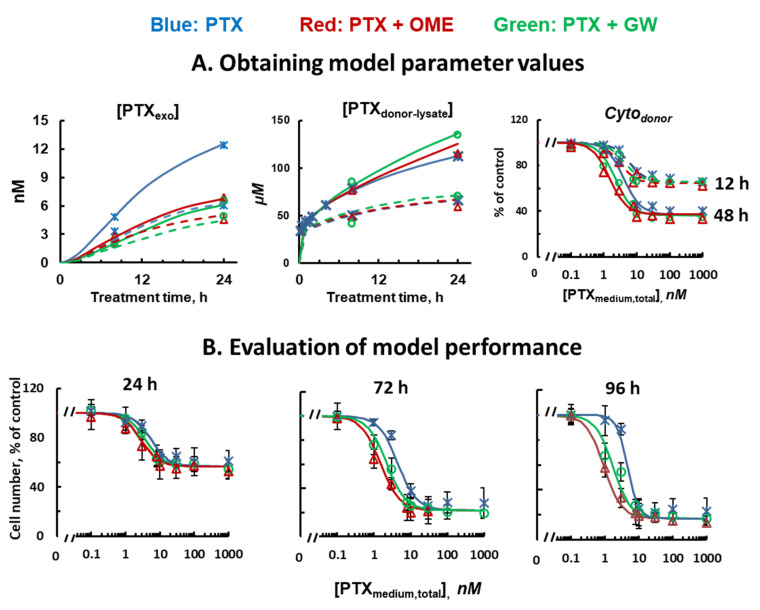
Model parameter estimation and model performance evaluation. (**A**) Model parameter estimation. Three PK and PD data sets obtained in MCF7 cells were used with the QP model (Equations (S2–S7) in Supplementary Materials) to estimate the best-fitting α and β values. Blue: single agent PTX. Red: PTX + OME pretreatment. Green: PTX + GW pretreatment. Left and middle panels: [PTX_exo_] vs. time and [PTX_donor-lysate_] vs. time, respectively. Note the different concentration units. 300 nM [PTX_medium,total_] (dotted curves) and 1000 nM [PTX_medium,total_] (solid curves). Right panel: *Cyto_donor_* vs. [PTX_medium,total_] for PTX treatments for 12 or 48 h. (**B**). Evaluation of QP model performance. Model-simulated *Cyto_donor_* of PTX treatments for 24, 72 and 96 h (curves) were compared to the experimental results (symbols; mean ± SEM, *n* = 3 experiments, duplicate samples per experiment); simulated results are within ±30% of observed mean values.

Figure 1 shows the QP model, comprising PK and PD components based on the known cellular PK and action mechanisms of PTX. MCF7 cells have low Pgp expression with negligible Pgp-mediated drug efflux; drug efflux is primarily via diffusion [29]. The model was fitted to all experimental, model-building data simultaneously to obtain the best-fitting α and β values (Figure 5A). α, which corresponded to the extent of inhibition of sorting PTX_cell,free_ into pre-exosome vesicles, equaled 0.61 ± 0.11 for OME and 0.33 ± 0.07 for GW, indicating OME was nearly twice as effective as GW in inhibiting formation of PTX_ves_ (*p* < 0.01). β, which corresponded to the extent of inhibition of release of EXO_PTX_, equaled 0.38 ± 0.17 for OME, and 0.47 ± 0.12 for GW, indicating GW was slightly more effective in inhibiting EXO_PTX_ release. The α and β values were then used to simulate treatment PD, which was dependent on both PTX concentrations and treatment durations; the simulated data were within the SEM of three experimentally obtained PD datasets (Figure 5B), indicating good model performance.

### 3.7. QP Model-Based Simulations to Quantify Non-Measurable PTX Entities and Intracellular Processes

**Figure 6:**
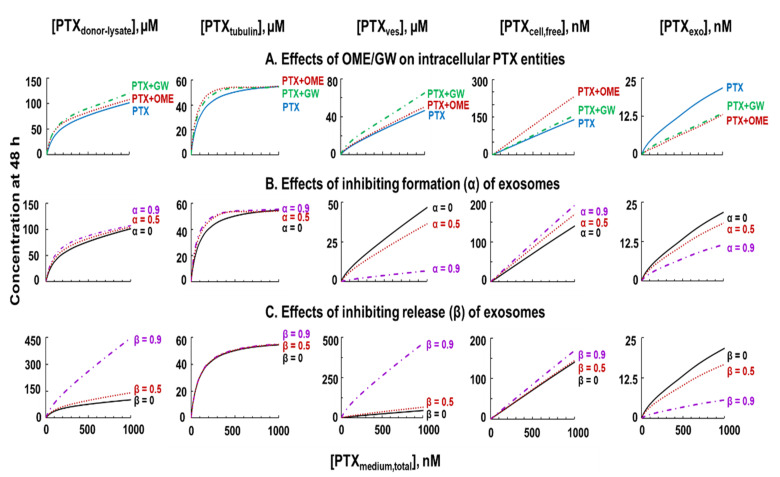
QP model-based simulations to depict changes in intracellular and extracellular PTX entities. Note the different *y*-axis scales. (**A**) Effects of OME and GW on concentrations of 5 PTX entities ([PTX_donor-lysate_], [PTX_tubulin_], [PTX_ves_], [PTX_cell,free_], [PTX_exo_]) as functions of extracellular [PTX_medium,total]_ (48 h treatment). Solid, single agent PTX; dashed, PTX + OME; dotted, PTX + GW. Note the different concentration units (µM for [PTX_donor-lysate_], [PTX_tubulin_] and [PTX_ves_], and nM for [_PTXcell,free_] and [PTX_exo_]). Note the plots of [PTX_donor-lysate_] and [PTX_exo_] included the experimental results obtained at 300 and 1000 nM [PTX_medium,total_] for quantitative comparison. (**B**) Effects of inhibiting exosome formation on intracellular PTX entities. α value was altered from 0 (i.e., single agent PTX without inhibitors, solid), 0.5 (50% inhibition, dotted), and 0.9 (90% inhibition, dashed). (**C**) Effects of inhibiting exosome exocytosis on intracellular PTX entities. β value was altered from 0 (solid), 0.5 (dotted), and 0.9 (dashed).

The validated QP model offered an opportunity to quantify the effects of altering individual intracellular processes that otherwise could not be measured. For example, we were able to measure only [PTX_donor-lysate_], which is the sum of three intracellular PTX entities instead of their individual values, and only [PTX_exo_], which is one of three extracellular PTX entities. In addition, quantification of [PTX_exo_] by the state-of-art LC-MS/MS was possible only at the two highest PTX concentrations of 300 and 1000 nM. Similarly, the live cell confocal microscopy studies, due to PTX-induced cytotoxicity and photobleaching, were limited to low PTX concentrations of 0.01 and 0.1 nM and the short treatment duration of 6 h. Hence, we used QP model-based simulations to examine the concentration-dependent kinetics of individual intracellular processes, at the full range of [PTX_medium,total_] of 0.01 to 1000 nM. Figure 6A shows the plots of 5 PTX entities ([PTX_donor-lysate_], [PTX_tubulin_], [PTX_ves_], [PTX_cell,free_], [PTX_exo_]) vs. [PTX_medium,total_] after 48 h PTX treatment. The plots of [PTX_donor-lysate_] and [PTX_exo_], for comparison, also included the experimental results obtained at 300 and 1000 nM [PTX_medium,total_], which overlapped with the simulated results.

The simulated results showed all intracellular entities increased with [PTX_medium,total_]. Changes in [PTX_tubulin_] followed Michaelis–Menten kinetics, as would be expected for saturable PTX binding to tubulin/microtubule, whereas changes in [PTX_ves_] and [PTX_cell,free_] increased linearly with [PTX_medium,total_], as would be expected for concentration-driven diffusion. In comparison, changes in [PTX_donor-lysate_] and [PTX_exo_] showed a combination of nonlinear increases at lower [PTX_medium,total_] followed by linear increases at higher [PTX_medium,total_]. Comparisons of the plots indicate GW pretreatment induced greater increases in [PTX_donor-lysate_] and [PTX_ves_] relative to OME, whereas OME pretreatment induced greater increases in [PTX_cell,free_].

Subsequently, we used the QP model to simulate the effects of inhibiting PTX-sorting into the pre-exosome vesicles vs. inhibiting exosome exocytosis on various PTX entities. Simulations were performed for no, medium, and high inhibition (α and β values of 0, 0.5 and 0.9, respectively); the results show increasing α or β had different effects on [PTX_ves_], [PTX_cell,free_], [PTX_exo_], and [PTX_donor-lysate_] (Figure 6B,C). For example, for treatment with 1000 nM PTX for 48 h, increasing α from 0 to 0.5 to 0.9, respectively, reduced [PTX_ves_] from 46 to 36 and 6.7 µM, increased [PTX_cell,free_] from 139 to 168 and 190 nM, and reduced [PTX_exo_] from 21 to 18 and 11 nM, whereas increasing β from 0 to 0.5 to 0.9, respectively, enhanced [PTX_ves_] from 46 to 66 and 459 µM, increased [PTX_cell,free_] from 139 to 143 and 169 nM, and reduced [PTX_exo_] from 21 to 16 and 6 nM.

**Figure 7:**
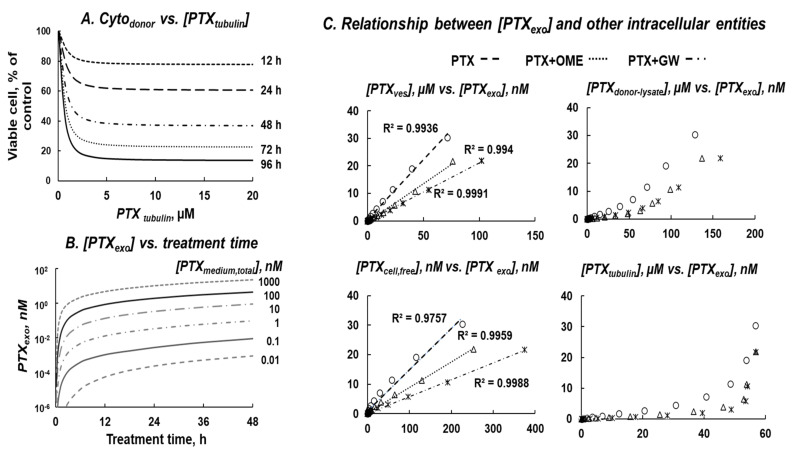
QP model-based simulations to evaluate PK and PD relationships between various intracellular PTX entities. (**A**) *Cyto_donor_* vs. [PTX_tubulin_]. (**B**) [PTX_exo_] as functions of [PTX_medium,total_] and treatment time. The respective area-under-curve values after 48 h treatments are 0.015, 0.164, 1.828, 17.70, 93.35, and 513.0 nM × h at [PTX_medium,total_] of 0.01, 0.1, 1, 10, 100, and 1000 nM. (**C**) Quantitative relationships between [PTX_exo_] and four intracellular entities ([PTX_donor-lysate_], [PTX_tubulin_], [PTX_ves_], [PTX_cell,free_]) in cells treated with 0.1 to 1000 nM PTX for 48 h, without or with OME (29 µM) and GW (10 µM). The respective r^2^ values for the regressed lines are >0.99 for [PTX_ves_], 0.98 to >0.99 for [PTX_cell,free_], and 0.85 to 0.95 for [PTX_donor-lysate_]. The relationships between [PTX_exo_] and [PTX_tubulin_] appeared biphasic with slower increases at lower [PTX_tubulin_] and higher increases at higher [PTX_tubulin_] (>50 µM).

Additional simulations were performed to examine PK and PD of various intracellular PTX entities. Figure 7A shows *E_obs,max_* of PTX *Cyto_donor_* linearly correlated with [PTX_tubulin_]. Figure 7B shows increased [PTX_exo_] with increasing treatment time and increasing [PTX_medium,total_]. Figure 7C shows [PTX_exo_] correlated linearly with [PTX_ves_] and [PTX_cell,free_] at all [PTX_medium,total_]. In contrast, the relationships between [PTX_exo_] and [PTX_tubulin_] appeared biphasic with slower increases at lower [PTX_tubulin_] and higher increases at higher [PTX_tubulin_] (>50 µM), likely due to saturation of tubulin binding at higher PTX concentrations (>50 µM). Additional calculations showed that, on average, [PTX_exo_] constituted <0.04% of [PTX_ves_] and <14% [PTX_cell,free_].

## 4. Discussion

We previously used experimental and computational studies to demonstrate that exosome is a mechanism of intercellular transfer with potential therapeutic importance (e.g., the amount of paclitaxel in exosomes was sufficient to cause pharmacology effects in the neighboring drug-naïve recipient cells) [10]. This earlier finding is of significance because the demonstration of this mechanism offers an explanation as to how paclitaxel is effective in treating solid tumors while it is well-established that paclitaxel, due to its extensive binding to cellular components, cannot readily penetrate the inner parts of a solid tumor. The current study extended our earlier model to include the effects of perturbing the sorting or release of exosomes on pharmacological outcomes, and offered the following new experimental and computational findings.

The experimental results reveal the following interesting findings on exosomes and effects of PTX, OME, and GW. First, the time-lapse live cell confocal microscopy and quantitative imaging studies using Lipoplex showed (a) relatively constant levels of extracellular exosomes suggesting a rapid equilibrium between the excretion and re-uptake of exosomes; and (b) PTX promoted, whereas OME inhibited, the appearance of extracellular exosomes (Figure 2). The latter was confirmed by the measurements of extracellular exosomes (Table 1). Second, the LC-MS/MS results indicate that EXO_PTX_ contained sufficient PTX to induce *Cyto_recip_* in drug-naïve cells. Third, pretreatment with either GW or OME abolished the PTX-enhanced extracellular exosomes (Figure 2, Table 1 and Table 2), indicating that these inhibitors of exosome formation and release abolished the PTX induction of exosomes. GW or OME pretreatment also reduced [PTX_exo_] and its *Cyto_recip_*, and enhanced [PTX_donor-lysate_] and *Cyto_donor_* of PTX. Fourth, OME and GW, on their own, reduced exosome production or secretion. In addition, drug interactivity analysis using two complementary methods (curve shift and UE) indicates synergy between PTX and OME or GW, indicating that the interactions between paclitaxel and exosome inhibitors had pharmacologically important effects. These experimental data are consistent with our earlier finding [10] that exosomes represent a therapeutically important efflux mechanism of PTX and that inhibition of exosome production or release can synergistically enhance PTX activity in exosome-donor cells but reduce PTX activity in exosome-recipient cells. In addition, the observed synergy between OME and PTX is consistent with the previous findings that OME enhances PTX activity in orthotropic human ovarian tumors as well as patient-derived xenografts, in immunodeficient mice [42], and that the (*S*)-enantiomer of OME enhances the effectiveness of docetaxel + cisplatin regimen in patients with triple negative metastatic breast cancer and extends the time-to-progression from 5.8 to 10.7 months [43]. Improved patient prognosis or sensitivity to taxane therapy upon combination with other proton pump inhibitors (e.g., esomeprazole and lansoprazole) were also reported in gastric cancer patients [44] and human melanoma or gastric adenocarcinoma xenografts [44,45].

The computational studies accounted for the multiple intracellular kinetic processes of PTX that are spatial dependent (e.g., binding to tubulin and processing by endocytic organelles occur only intracellularly) and often nonlinear (e.g., saturable tubulin binding). We have shown the PK/PD complexities of PTX confounded data interpretation, and have developed several computational models to elucidate PTX intracellular PK [10,27,29] that have since been adopted by several investigator groups [46,47,48,49]. Our current quantitative cellular scale model of exosome-mediated intercellular paclitaxel transfer, established using PK and PD data of PTX without or with OME or GW pretreatment, was validated with additional PD data. Simulations using the validated QP model revealed the quantitative relationships between [PTX_exo_] and 3 non-measurable intracellular PTX entities ([PTX_cell,free_], [PTX_tubulin_], [PTX_ves_]), the kinetics of [PTX_exo_] as functions of [PTX_medium,total_] and treatment time, and the nonlinear increases in [PTX_tubulin_] by OME/GW pretreatment due to saturation of tubulin binding. The simulation results further indicate that while inhibiting the sorting of PTX into PTXves and inhibiting the release of EXOPTX both lowered [PTXexo] and [PTXcell,free], these two inhibitors affected the intracellular and extracellular paclitaxel entities differently, yielded opposite changes in [PTX_ves_], and exerted different effects on intracellular PTX processing and distribution in subcellular compartments.

To our knowledge, our earlier quantitative cellular scale model of exosome-mediated intercellular paclitaxel transfer [10], and the current model, which included the effects of perturbations of exosome sorting and release, are the first of its kind. It is noted that quantitative systems pharmacology modeling is an emerging field that has been identified by both NIH and FDA as a potentially useful tool to improve the effectiveness and efficiency of the development of new therapeutics [50,51,52]. The current study represents an example of successful use of QP modeling to interrogate the intracellular events that could not be measured using the currently available experimental technologies. We propose that the current model can be used as a template for the future development of exosomes as a drug carrier system. For example, linking this cellular scale model with other scale models (e.g., whole body scale, organ scale) will enable an investigator to determine the target site delivery and retention of the candidate exosome.

## 5. Conclusions

In summary, the experimental results of this study demonstrated that perturbations of exosome sorting or release quantitatively accounted for the synergy between PTX and OME or GW. Using these results together with QP modeling further provided qualitative and quantitative information on the intracellular processing of PTX-loaded exosomes that otherwise could not be readily measured. In view of the increasing interests in using engineered exosomes as carriers of various types of therapeutics, e.g., small molecule drugs, proteins and nucleic acids, additional studies to elucidate the intracellular fates of exosomes are warranted.

## Figures and Tables

**Table 1:** Effects of PTX, OME, and GW on exosome production or release. Cells were treated with OME (29 µM), GW (10 µM), or PTX (100 nM) for 24 h. For combinations of OME + PTX or GW + PTX, cells were pretreated with either OME or GW, followed by treatment with PTX. Conditioned Medium was collected, and the number of exosomes were determined by analyzing the AChE activity and a simultaneously prepared standard curve. Control cells were similarly processed but had no drug treatment. Mean ± SEM (*n* = 3 experiments, triplicates per experiment).

Cell	Exosome Recovered in Conditioned Medium					
	No Drug, Number/10^6^ Cells	% Change from Control				
		+PTX	+OME	+GW	+OME + PTX	+GW + PTX
LM2	362 ± 29	69 ± 6.4 *	−26 ± 3.9 *	−26 ± 4.0 *	−11 ± 2.0 **	−30 ± 7.7 *
MCF7	266 ± 17	43 ± 6.3 *	−43 ± 6.0 *	−21 ± 2.4 *	−12 ± 3.2 **	−26 ± 7.4 *
A2780	227 ±39	55 ± 3.1 *	−1.9 ± 3.7	−9.1 ± 2.7	−6.9 ± 4.1	−12 ± 4.5
OVCAR4	265 ± 23	50 ± 2.9 *	−4.8 ± 2.5	−9.6 ± 1.1	−0.9 ± 4.2	−2.5 ± 2.1

* *p* < 0.05 vs. control, ** *p* < 0.05 compared to single agent OME (unpaired, two-tailed Student’s *t*-test).

**Table 2:** Effect of OME and GW pretreatment on [PTX_exo_] and [PTX_donor-lysate_]. Exosome-donor cells were pretreated with OME (29 µM) or GW (10 µM) for 24 h, followed by treatment with 1000 nM PTX for 24 h. Exosomes and cell lysates were analyzed for PTX levels using LC-MS/MS. Control cells were similarly processed but had no OME or GW pretreatment. A. Different cells treated with PTX 1000 nM for 48 h. B. MCF7 cells treated with 300 and 1000 nM [PTX_medium,total_] for 8 and 24 h. Mean ± SEM (3 experiments, triplicates per experiment).

		pmol/10^6^ Donor Cells		% Change Compared to Single Agent PTX			
				OME + PTX		GW + PTX	
		[PTX_exo_]	[PTX_donor-lysate_]	[PTX_exo_]	[PTX_donor-lysate_]	[PTX_exo_]	[PTX_donor-lysate_]
Cell		A. Different cells treated with 1000 nM PTX for 24 h					
LM2		6 ± 1	32 ± 1	−25 ± 17	+34 ± 8.1	−79 ± 13 *	+66 ± 20 *
MCF7		12 ± 1	76 ± 7	−42 ± 9 *	+15 ± 8	−49 ± 5 *	+23 ± 8
A2780		5 ± 1	52 ± 6	−22 ± 2	+131± 9 *	−34 ± 8 *	+105 ± 7 *
OVCAR4		7 ± 2	72 ± 6	−45 ± 5 *	+26 ± 10	−74 ± 7 *	+97 ± 3 *
[PTX_medium,total_]		B. MCF7 cells treated with 300 or 1000 nM PTX for 8 or 24 h					
300 nM	8 h	21 ± 9	34 ± 13	−36 ± 18 *	−4 ± 11	−44 ± 14 *	+19 ± 9
	24 h	11 ± 6	45 ± 2	−25 ± 13	+11 ± 7	−19 ± 6	+7 ± 12
1000 nM	8 h	19 ± 8	53 ± 9	−41 ± 17 *	+3 ± 5	−54 ± 19 *	+8 ± 3
	24 h	12 ± 1	76 ± 7	−42 ± 9 *	+15 ± 8	−49 ± 5 *	+23 ± 8

* *p* < 0.05 vs. single agent PTX (unpaired, two-tailed Student’s *t*-test).

## Data Availability

All relevant data are contained in the article.

## Supplementary Materials

The following are available online at https://www.mdpi.com/article/10.3390/pharmaceutics13070997/s1, Table S1: PTX cellular pharmacokinetic and pharmacodynamics model parameters.
