# Supplementary material for: A Quantitative Pharmacology Model of Exosome-Mediated Drug Efflux and Perturbation-Induced Synergy
Source: Pharmaceutics. 2021 Jun 30;13(7):997. doi: 10.3390/pharmaceutics13070997 (PMC8308960; doi:10.3390/pharmaceutics13070997)
Supplement: Supplementary file 1 [file pharmaceutics-13-00997-s001.zip › pharmaceutics-1226308-supplementary.pdf]

pharmaceutics

MDPI

Supplementary Materials: A Quantitative Pharmacology Model of Exosome-Mediated Drug Efflux and Perturbation-Induced Synergy

Jin Wang, Bertrand Z. Yeung, M. Guillaume Wientjes, Minjian Cui, Cody J. Peer, Ze Lu, William D. Figg, Sukyung Woo and Jessie L.-S. Au

1. Quantitative Pharmacology-Pharmacokinetic Model

The QP model depicted in Figure 4 comprises two components, PK and PD. The PK component describes the changes of PTX concentrations as functions of binding to tubulin, sorting into exosomes, and efflux by exosomes; the model parameters are presented in Table S2A. The PD component describes the PTX cytotoxicity as a function of tubulin-bound PTX concentration; the model parameters are explained in Table S2B. The model assumptions are (a) unbound drug in medium enters cells by passive diffusion (D_{fd}); (b) saturable drug binding to proteins in extracellular fluid ($B_{medium,max}$, $Kd_{medium,bound}$) and to intracellular tubulin ($B_{tubulin,max}$, $k_{tubulin,on}$, $k_{tubulin,off}$); (c) drug efflux from cells uses a combination of passive diffusion (D_{fd}), Pgp-mediated saturable efflux ($Jmax_{Pgp}$, Kd_{Pgp}), and first-order release of drug-containing exosomes ($k_{release}$); (d) first-order sorting of intracellular unbound drug and tubulin-bound drug into exosomes, $k_{formation,free}$ and $k_{formation,tubulin}$ respectively through the endosomal transport system; and (e) internalization of drug-containing exosomes through saturable receptor-mediated endocytosis ($Jmax_{inter,exo}$, $Kd_{inter,exo}$). The effects of OME and GW are incorporated into the model as inhibitors of PTX sorting into exosomes (α) and exosome release (β); their values were between zero (no inhibition) and 1 (complete inhibition). Definitions of drug concentrations, transport and binding parameters, and exosome-related parameters are shown in Figure 4 legends.

Equations (S1) and (S2) depict [$PTX_{donor\text{-}lysate}$] and [$PTX_{medium,total}$]. Equations (S3)–(S5) describe the time-dependent changes of [$PTX_{cell,free}$], [PTX_{ves}], and [$PTX_{tubulin}$]. Equation (S6) describes the changes of $B_{tubulin,max}$ (μM) with time (h) and [$PTX_{cell,free}$] (nM). Equations (S7)–(S9)describe changes of [$PTX_{medium,free}$], [PTX_{exo}], and [$PTX_{medium,bound}$] with time.

$$[PTX_{donor-lysate}] = [PTX_{cell,free}] + [PTX_{ves}] + [PTX_{tubulin}] \quad \text{(S1)}$$

$$[PTX_{medium,total}] = [PTX_{medium,free}] + [PTX_{medium,bound}] + [PTX_{exo}] \quad \text{(S2)}$$

$$\frac{d[PTX_{cell,free}]}{dt}$$

$$= \frac{\left(D_{fd} \cdot [PTX_{medium,free}] - D_{fd} \cdot [PTX_{cell,free}] - \frac{Jmax_{Pgp} \cdot [PTX_{cell,free}]}{Kd_{Pgp} + [PTX_{cell,free}]} + \frac{Jmax_{inter,exo} \cdot [PTX_{e} [illegible]}{Kd_{inter,exo} + [PTX_{e} [illegible]}\right.}{V_{cell}} \quad \text{(S3)}$$

$$- \alpha) \cdot k_{formation,free} \cdot [PTX_{cell,free}] + k_{tubulin,off} \cdot [PTX_{tubulin}]$$

$$- k_{tubulin,on} \cdot [PTX_{cell,free}] \cdot (B_{tubulin,max} - [PTX_{tubulin}])$$

$$\frac{d[PTX_{ves}]}{dt} = (1 - \alpha) \cdot (k_{formation,free} \cdot [PTX_{cell,free}] + k_{formation,tubulin} \cdot [PTX_{tubulin}]) - k_{release} \cdot (1 - \beta) \cdot [PTX_{ves}] \quad \text{(S4)}$$

$$\frac{d[PTX_{tubulin}]}{dt} = k_{tubulin,on} \cdot [PTX_{cell,free}] \cdot (B_{tubulin,max} - [PTX_{tubulin}]) - k_{tubulin,off} \cdot [PTX_{tubulin}] - (1-\alpha) \cdot k_{formation,tubulin} \cdot [PTX_{tubulin}] \quad \text{(S5)}$$

$$B_{tubulin,max} = 59.2 + 0.408 \cdot [PTX_{cell,free}]^{0.2941} \cdot time \quad \text{(S6)}$$

$$\frac{d[PTX_{medium,free}]}{dt} = \left(-D_{fd} \cdot [PTX_{medium,free}] + D_{fd} \cdot [PTX_{cell,free}] + \frac{J_{max,Pgp} \cdot [PTX_{cell,free}]}{Kd_{Pgp} + [PTX_{cell,free}]}\right) \cdot \frac{TCN}{V_{medium}} \quad \text{(S7)}$$

$$\frac{d[PTX_{exo}]}{dt} = ((1-\beta) \cdot k_{release} \cdot [PTX_{ves}] \cdot V_{cell} - \frac{Jmax_{inter,exo} \cdot [PTX_{exo}]}{Kd_{inter,exo} + [PTX_{exo}]}) \cdot \frac{TCN}{V_{medium}} \quad \text{(S8)}$$

$$[PTX_{medium,bound}] = \frac{[PTX_{medium,free}] \cdot B_{medium,max}}{Kd_{medium,bound} + [PTX_{medium,free}]} \quad \text{(S9)}$$

For initial conditions (before treatment), $[PTX_{exo}]$ equals zero. $[PTX_{medium,free}]$ and $[PTX_{medium,bound}]$ were calculated from $[PTX_{medium,total}]$ using Equation (S10) (obtained by substituting Equation (S9) into Equation (S2), followed by rearrangement).

$$[PTX_{medium,free}] = \frac{-(Kd_{medium,bound} + B_{medium,max} - [PTX_{medium,total}]) + \sqrt{\begin{matrix}(Kd_{medium,bound} + B_{medium,max} - [PTX_{medium,tot} \\ 4 \cdot ([PTX_{medium,total}] \cdot Kd_{medium,bound})\end{matrix}}}{2} \quad \text{(S10)}$$

2. Quantitative Pharmacology-Pharmacodynamic Model

For the PD component of the QP model, *ICN* and *TCN* are respectively the initial and total cell number, and V_{cell} and V_{medium} are respectively the volume of a single cell and extracellular medium; these parameters were experimentally determined or calculated. The PD model describes changes in *TCN* as a function of drug treatment (concentration and time). Equation (S11) depicts the net change in *TCN* due to (a) cell growth over time at a rate constant k_{growth} until confluence, and (b) drug-induced cell kill as a function of concentration of tubulin-bound drug. *TCNss* is maximal cell number at confluence. k_{kill} is maximum cell kill rate constant. EC_{50} is tubulin-bound drug concentration that generates 50% maximum cell kill, *n* is the Hill exponent of PTX cytotoxicity. Equation (S12) depicts the time-dependent changes in EC_{50} where $EC_{50,initial}$ is the EC_{50} value at time zero, and γ_{EC50} is the rate of EC_{50} change per unit time *t*. These PD parameters were fixed as the same values in our previous publication (11).

$$\frac{dTCN}{dt} = k_{growth} \cdot \text{TCN} \cdot \left(1 - \frac{TCN}{TCNss}\right) - k_{kill} \cdot \left(\frac{[PTX_{tubulin}]^n}{[PTX_{tubulin}]^n + EC_{50}^n}\right) \cdot \text{TCN} \quad \text{(S11)}$$

$$EC_{50} = EC_{50,initial} + \gamma_{EC50} \cdot t \quad \text{(S12)}$$

Table S1. PTX cellular pharmacokinetic and pharmacodynamics model parameters**.** Some parameter values were obtained from the literature and some were obtained from analyzing the experimental results of the current study (indicated as Analysis). Mean values of parameters obtained from Analysis are shown with % CV in parentheses.

Category	Parameter	Annotation	Value (% CV)	Source
		A. Cellular pharmacokinetic model		
Pgp-mediated efflux	$Jmax_{pgp}$ (pmole·h⁻¹·cell⁻¹)	Maximum Pgp efflux rate	2.8×10^{-6}	(20)
	Kd_{pgp} (nM)	Dissociation constant of drug from Pgp	13.9	(20)
Diffusion	D_{fd} (μL·h⁻¹·cell⁻¹)	Diffusion rate constant of free PTX across cell membrane	3.34×10^{-3}	(12)
Intracellular microtubule binding	$k_{tubulin,off}$ (s^{-1})	Rate constant of PTX dissociation from tubulin	30	(36)
	$k_{tubulin,on}$ ($nM^{-1}\cdot s^{-1}$)	Rate constant of PTX association with tubulin	2	(36)
	NSB	Proportionality constant for nonsaturable binding in cells	0.148	(20)
Binding to extracellular fluid	$B_{medium,max}$ (μM)	Maximal extracellular drug binding sites	3.94	(20)
	$Kd_{medium,bound}$ (nM)	Dissociation constant of PTX from extracellular binding sites	781	(20)
	V_{cell} (μL)	Volume of single cell	2.06×10^{-6}	(12)
	V_{medium} (μL)	Medium volume	1000	Assigned
	NSB	Nonsaturable binding	0	
Exosome formation, release and internalization	$Jmax_{,inter,exo}$ (pmole·h⁻¹·cell⁻¹)	Maximum rate of endocytosis of extracellular exosomes	0.038	(11)
	$Kd_{inter,exo}$ (nM)	Dissociation constant of exosomes from endocytosis receptor	63	(11)
	$k_{formation,free}$ (h^{-1})	Rate constant for sorting intracellular $PTX_{cell,free}$ into exosome	31	(11)
	$k_{formation,tubulin}$ (h^{-1})	Rate constant for sorting intracellular $PTX_{tubulin}$ into exosome	0.02 (58)	Estimated
	$k_{release}$ (h^{-1})	Rate constant for exosomes release into extracellular fluid	0.105	(11)
	α	Extent of inhibition of exosome formation	GW: 0.33 (21)<br>OME: 0.61 (18)	Estimated
	β	Extent of inhibition of exosome release	GW: 0.47 (26)<br>OME: 0.38 (45)	Estimated
		B. Pharmacodynamic model		
Drug cytotoxicity	k_{growth} (h^{-1})	Cell growth rate constant	0.0288	(11)
	k_{kill} (h^{-1})	Maximum cell kill rate constant	0.0183	(11)
	$EC_{50,initial}$ (nM)	Tubulin-bound concentration to produce 50% $E_{obs,max}$ at time zero	826	(11)
	γ_{EC50} (nM·h⁻¹)	Change in IC_{50} over treatment time	7.2	(11)
	n	Hill exponent of PTX concentration-cytotoxicity curve	1.67	(11)
	TCN_{ss}	Maximum cell number at confluence	1.1×10^{6}	Measured
	ICN	Initial cell number	5×10^{3}	Assigned

3. Macro Plug-in Script that was Written to Automatically Run the Multi-step Fluorescence Signal Quantification Through ImageJ (National Institutes of Health, MD)

```
//This macro demonstrates how the living cell confocal images were processed and how
    extracellular endosome-related vesicles fluorescence signals were quantified to produce the
    plots in Figure 2B.

//First, read the original confocal image file (named "Crop001_t04_z4.jpg" in this example).
open("~Crop001_t04_z4.jpg");

//Extract the text string of directory and title of this image file so the output files can be properly
    named after processing.
Dir=getDirectory("image");
Title=getTitle();
Prefix = substring (Title, 0, lastIndexOf(Title,"."));
```

```
//The original confocal image has three pseudo channels (green, red and blue), which have to be
    separated. Then the green channel, which represents Rab7-GFP staining, can be closed to
    focus the following analysis on red (Rab5 RFP) and blue (AF647 conjugated siRNA)
    channels.
run("Split Channels");
selectWindow(Prefix+".jpg (green)");
close();

// Adjust the contrast threshold of red channel (Rab5 RFP) to amplify the intracellular area signal
    without creating too much background signal in the extracellular area. This threshold was
    empirically selected and fixed for all the images that were processed in current study.
selectWindow(Prefix+".jpg (red)");
setAutoThreshold("Default");
setThreshold(12, 255);
run("Convert to Mask");

// Similarly, the contrast threshold of red channel (Rab5 RFP) to amplify the extracellular area
    signal without creating too much background signal in the intracellular area. This threshold
    was empirically selected and fixed for all the images that were processed in current study.
selectWindow(Prefix+".jpg (blue)");
setAutoThreshold("Default");
setThreshold(30, 255);
run("Convert to Mask");

// Next, ImageJ tool "Image Calculator" was utilized to identify the area where red (Rab5 RFP)
    and blue (siRNA) signal co-localized through function "multiply". Since most of the red
    (Rab5 RFP) was intracellular while all the blue signal stayed in extracellular area, the
    resulted co-localization particles represented the endosome-related vesicles that were bubbled
    out to the extracellular matrix.
imageCalculator("Multiply create", Prefix+".jpg (red)",Prefix+".jpg (blue)");
saveAs("Jpeg", Dir+Prefix+"_multiply");

// Then the intensity of red and blue signal co-localized area was quantified and recorded.
run("Analyze Particles...", "size=4-Infinity circularity=0.0-1.00 display clear add");
run("Clear Results");
run("Close All");

// Lastly, the overall intensity of blue signal was quantified as the background level in order to
    normalize the quantified red-blue colocalization intensity.
open(Dir+Title);
run("Split Channels");
selectWindow(Prefix+".jpg (green)");
close();
selectWindow(Prefix+".jpg (blue)");
roiManager("Measure");
selectWindow("Results");
saveAs("Text", Dir+Prefix+"_blue"+".csv");

// Remember to clear up the results in cache and close all the windows so the program can be set
    up to analyze the next image.
run("Clear Results");
roiManager("Delete");
run("Close All")
```
